# *catena*-Poly[[tri­aqua­magnesium]-μ_2_-malonato]

**DOI:** 10.1107/S1600536813034193

**Published:** 2013-12-24

**Authors:** Tim de Klijn, Martin Lutz

**Affiliations:** a Bijvoet Center for Biomolecular Research, Crystal and Structural Chemistry, Faculty of Science, Utrecht University, Padualaan 8, 3584 CH Utrecht, The Netherlands

## Abstract

In the title compound, [Mg(C_3_H_2_O_4_)(H_2_O)_3_]_*n*_, the metal atom is in an octa­hedral environment. The octa­hedra are connected by malonate anions, forming chains along the *c-*axis direction. O—H⋯O hydrogen bonds link these chains into a three-dimensional network.

## Related literature   

For related divalent metal malonates, see: Walter-Levy *et al.* (1973 ▶); Ray & Hathaway (1982 ▶); Delgado *et al.* (2004 ▶); Zheng & Xie (2004 ▶). For the synthesis, see: Delgado *et al.* (2004 ▶). For the geometry of coordinating water mol­ecules, see: Ptasiewicz-Bak *et al.* (1999 ▶). For the determination of the mol­ecular symmetry, see: Pilati & Forni (1998 ▶). For ring puckering analysis, see: Evans & Boeyens (1989 ▶).
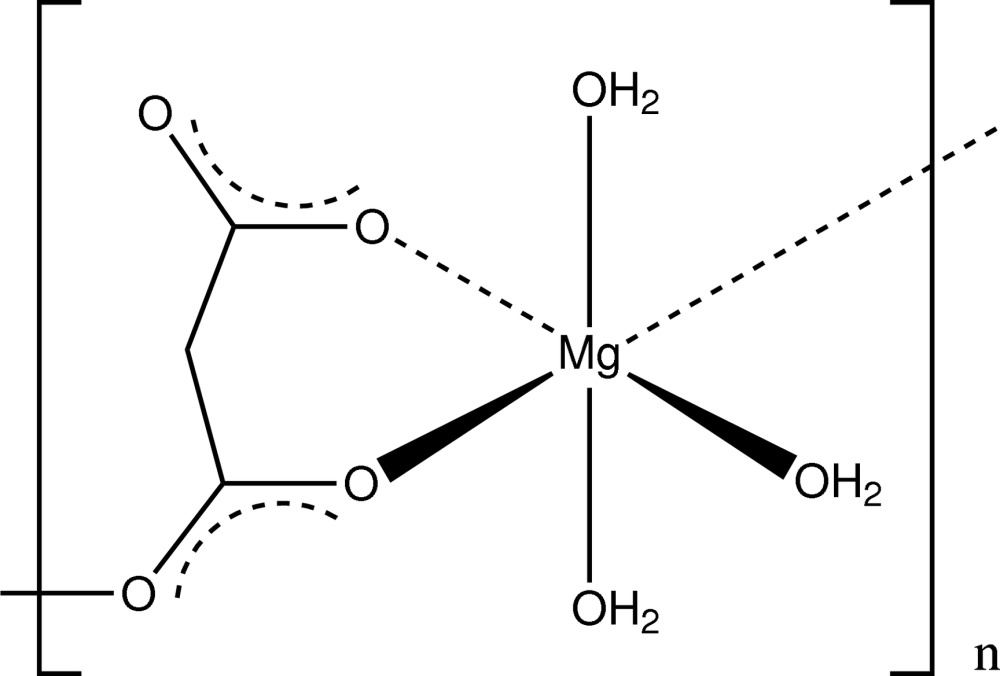


## Experimental   

### 

#### Crystal data  

[Mg(C_3_H_2_O_4_)(H_2_O)_3_]
*M* *_r_* = 180.40
Orthorhombic, 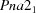

*a* = 19.8109 (15) Å
*b* = 5.9314 (4) Å
*c* = 6.0920 (4) Å
*V* = 715.84 (9) Å^3^
*Z* = 4
Mo *K*α radiation
μ = 0.24 mm^−1^
*T* = 150 K
0.51 × 0.23 × 0.07 mm

#### Data collection  

Bruker Kappa APEXII diffractometer
Absorption correction: multi-scan (*SADABS*; Sheldrick, 2012 ▶) *T*_min_ = 0.618, *T*_max_ = 0.746
7334 measured reflections
1603 independent reflections
1533 reflections with *I* > 2σ(*I*)
*R*_int_ = 0.023

#### Refinement  

*R*[*F* ^2^ > 2σ(*F* ^2^)] = 0.023
*wR*(*F* ^2^) = 0.061
*S* = 1.10
1603 reflections
124 parameters
1 restraint
H atoms treated by a mixture of independent and constrained refinement
Δρ_max_ = 0.25 e Å^−3^
Δρ_min_ = −0.22 e Å^−3^
Absolute structure: Flack parameter determined using 674 quotients [(*I* ^+^)−(*I* ^−^)]/[(*I* ^+^)+(*I* ^−^)] (Parsons *et al.*, 2013 ▶)
Absolute structure parameter: 0.00 (9)

### 

Data collection: *APEX2* (Bruker, 2007 ▶); cell refinement: *Peakref* (Schreurs, 2013 ▶); data reduction: *Eval15* (Schreurs *et al.*, 2010 ▶) and *SADABS* (Sheldrick, 2012 ▶); program(s) used to solve structure: *SHELXT* (Sheldrick, 2008 ▶); program(s) used to refine structure: *SHELXL2013* (Sheldrick, 2008 ▶); molecular graphics: *PLATON* (Spek, 2009 ▶) and *DRAWxtl* (Finger *et al.*, 2007 ▶); method used to prepare material for publication: manual editing of the *SHELXL* output.

## Supplementary Material

Crystal structure: contains datablock(s) I, global. DOI: 10.1107/S1600536813034193/kj2236sup1.cif

Structure factors: contains datablock(s) I. DOI: 10.1107/S1600536813034193/kj2236Isup2.hkl

Additional supporting information:  crystallographic information; 3D view; checkCIF report

## Figures and Tables

**Table 1:** Selected bond lengths (Å)

Mg1—O3^i^	2.0323 (18)
Mg1—O5	2.0377 (15)
Mg1—O4	2.0700 (16)
Mg1—O6	2.0706 (15)
Mg1—O1	2.0725 (15)
Mg1—O7	2.1273 (14)

Symmetry code: (i) 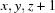.

**Table 2:** Hydrogen-bond geometry (Å, °)

*D*—H⋯*A*	*D*—H	H⋯*A*	*D*⋯*A*	*D*—H⋯*A*
O5—H1*O*⋯O2^ii^	0.91 (3)	1.80 (3)	2.701 (2)	170 (3)
O5—H2*O*⋯O2^iii^	0.85 (4)	1.85 (4)	2.678 (2)	165 (3)
O6—H3*O*⋯O4^iv^	0.83 (3)	1.93 (3)	2.759 (2)	175 (3)
O6—H4*O*⋯O7^v^	0.73 (4)	2.11 (4)	2.838 (2)	177 (3)
O7—H5*O*⋯O3^vi^	0.86 (3)	2.11 (3)	2.963 (2)	172 (3)
O7—H6*O*⋯O1^iii^	0.78 (3)	1.93 (3)	2.703 (2)	169 (3)

Symmetry codes: (ii) 
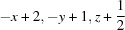
; (iii) 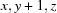; (iv) 
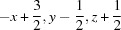
; (v) 
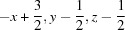
; (vi) 
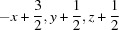
.

**Fig. 1.**
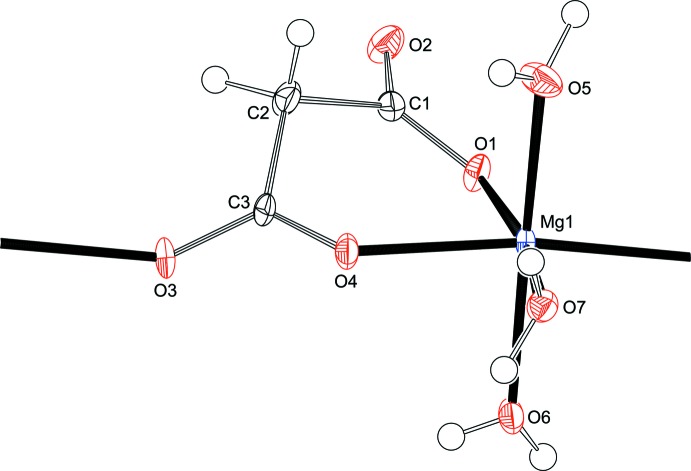
Asymmetric unit in the crystal structure of title compound. View along the b axis. Displacement ellipsoids are drawn at the 50% probability level. H atoms are drawn as small spheres of arbitrary radii.

**Fig. 2.**
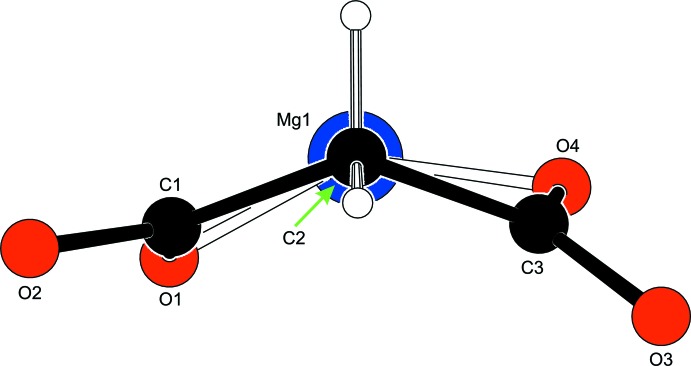
Puckering of the six-membered chelate ring obtained by the coordination of the malonate dianion to the Mg(II) cation.

**Fig. 3.**
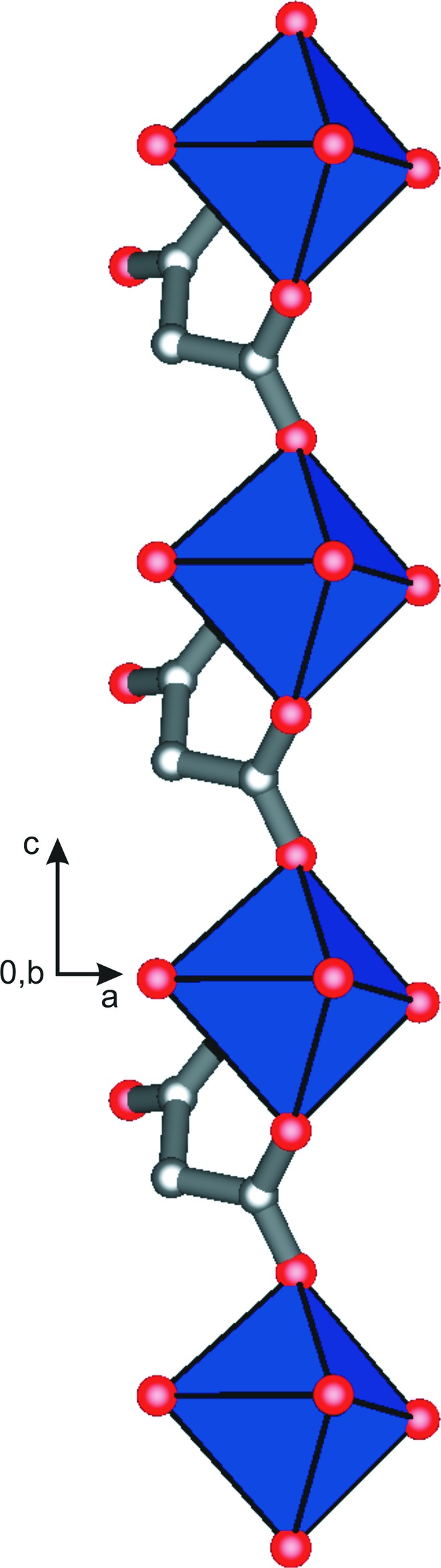
One-dimensional coordination chain in the direction of the c axis.

## supplementary crystallographic information

### 1. Comment

The malonates of the divalent metals Zn, Ni, and Co are isostructural and crystallize as dihydrates in the monoclinic space group C2/m (Walter-Levy *et al.*, 1973; Ray & Hathaway, 1982; Delgado *et al.*, 2004; Zheng & Xie, 2004). The metals are located on sites with 2/m symmetry, octahedrally surrounded by six O atoms. The water molecules and the malonate ligand have mirror symmetry. Overall, this leads to a two-dimensional coordination network in which the layers are interlinked by O—H···O hydrogen bonds.

In this context we set out to synthesize the corresponding Mg(II) complex. Interestingly, the title compound is not isostructural to the above mentioned Zn, Ni, and Co complexes but crystallizes as a trihydrate in the orthorhombic space group Pna2_1_. All atoms are located on general positions without symmetry. The magnesium centers are surrounded by six O atoms in a slightly distorted octahedral geometry. Three O atoms are from the deprotonated malonate ligand, and three O atoms are from the coordinated water molecules (Figure 1). The Mg—O distance to O7 is the largest. O7 is donor of two and acceptor of one hydrogen bond. Water O atoms O5 and O6 do not accept hydrogen bonds. According to the definition by Ptasiewicz-Bak *et al.* (1999), water molecule O5 is trigonally coordinated, and water molecules O6 and O7 in tetrahedral direction. The angles between the planes of the water molecules and the O—Mg bonds are 7(4), 29 (3), and 42 (3)° for O5, O6, and O7, respectively.

While the malonate dianion has no crystallographic symmetry, it still has an approximate mirror symmetry with an r.m.s. deviation of 0.20 Å (Pilati & Forni, 1998). By coordination to the Mg, a six-membered chelate ring is formed (Figure 2). According to the algorithm by Evans & Boeyens (1989), the conformation of the ring can be described as linear combination of 75% boat, 23% twist-boat, and 2% chair conformation.

In the title compound, the malonate dianion acts as a bridging ligand, which connects the Mg octahedra into a one-dimensional chain in the direction of the *c* axis (Figure 3). This distance between the Mg centers is consequently the length of the *c* axis [6.0920 (4) Å]. The one-dimensional coordination chains are linked by O—H···O hydrogen bonds into a three-dimensional network (Table 2). All three water molecules act as hydrogen bond donors. The non-coordinated O2 accepts two hydrogen bonds. Each of the coordinated O atoms O1, O3, and O4 accept one hydrogen bond, respectively, and one hydrogen bond is accepted by the water molecule at O7.

### 2. Experimental

Crystals were prepared according to the method by Delgado *et al.* (2004). 2.15 g (10.0 mmol) of magnesium acetate tetrahydrate (Fluka) were dissolved in 20 ml water. This solution was slowly added to a solution of 1.16 g (11.1 mmol) of malonic acid (Fluka) in 20 ml water. The resulting mixture was concentrated by evaporation at 333 K and normal pressure. After standing at room temperature over night, the crystals were obtained.

### 3. Refinement

The crystal consisted of two fragments and was consequently integrated with two orientation matrices. The two matrices are related by a rotation angle of 8.6 ° about an axis approximately collinear with the *c* axis. Only the non-overlapping reflections were used for the structure refinement.

### Crystal data

**Table:**

[Mg(C_3_H_2_O_4_)(H_2_O)_3_]	*D*_x_ = 1.674 Mg m^−^^3^
*M**_r_* = 180.40	Mo *K*α radiation, λ = 0.71073 Å
Orthorhombic, *P**n**a*2_1_	Cell parameters from 6736 reflections
*a* = 19.8109 (15) Å	θ = 2.1–27.6°
*b* = 5.9314 (4) Å	µ = 0.24 mm^−^^1^
*c* = 6.0920 (4) Å	*T* = 150 K
*V* = 715.84 (9) Å^3^	Irregular block, colourless
*Z* = 4	0.51 × 0.23 × 0.07 mm
*F*(000) = 376	

### Data collection

**Table:**

Bruker Kappa APEXII diffractometer	1533 reflections with *I* > 2σ(*I*)
Radiation source: sealed tube	*R*_int_ = 0.023
φ and ω scans	θ_max_ = 27.5°, θ_min_ = 2.1°
Absorption correction: multi-scan (*SADABS*; Sheldrick, 2012)	*h* = −25→25
*T*_min_ = 0.618, *T*_max_ = 0.746	*k* = −7→7
7334 measured reflections	*l* = −7→7
1603 independent reflections	

### Refinement

**Table:**

Refinement on *F*^2^	Secondary atom site location: difference Fourier map
Least-squares matrix: full	Hydrogen site location: difference Fourier map
*R*[*F*^2^ > 2σ(*F*^2^)] = 0.023	H atoms treated by a mixture of independent and constrained refinement
*wR*(*F*^2^) = 0.061	*w* = 1/[σ^2^(*F*_o_^2^) + (0.0408*P*)^2^ + 0.0476*P*] where *P* = (*F*_o_^2^ + 2*F*_c_^2^)/3
*S* = 1.10	(Δ/σ)_max_ = 0.001
1603 reflections	Δρ_max_ = 0.25 e Å^−^^3^
124 parameters	Δρ_min_ = −0.22 e Å^−^^3^
1 restraint	Absolute structure: Flack parameter determined using 674 quotients [(*I*^+^)-(*I*^-^)]/[(*I*^+^)+(*I*^-^)] (Parsons *et al.*, 2013)
Primary atom site location: heavy-atom method	Absolute structure parameter: 0.00 (9)

### Special details

**Table:**

Geometry. All e.s.d.'s (except the e.s.d. in the dihedral angle between two l.s. planes) are estimated using the full covariance matrix. The cell e.s.d.'s are taken into account individually in the estimation of e.s.d.'s in distances, angles and torsion angles; correlations between e.s.d.'s in cell parameters are only used when they are defined by crystal symmetry. An approximate (isotropic) treatment of cell e.s.d.'s is used for estimating e.s.d.'s involving l.s. planes.

### Fractional atomic coordinates and isotropic or equivalent isotropic displacement parameters (Å^2^)

**Table:**

	*x*	*y*	*z*	*U*_iso_*/*U*_eq_	
Mg1	0.83901 (3)	0.75469 (12)	0.46114 (12)	0.01041 (16)	
O1	0.87901 (7)	0.4456 (2)	0.3702 (2)	0.0147 (3)	
O2	0.95295 (7)	0.2264 (2)	0.1994 (3)	0.0191 (3)	
O3	0.83004 (7)	0.6848 (3)	−0.2136 (3)	0.0169 (3)	
O4	0.83378 (6)	0.8126 (3)	0.1265 (2)	0.0145 (3)	
O5	0.93242 (7)	0.8953 (3)	0.4907 (3)	0.0213 (3)	
H1O	0.9704 (15)	0.838 (5)	0.554 (6)	0.038 (7)*	
H2O	0.9365 (14)	1.016 (6)	0.417 (6)	0.037 (8)*	
O6	0.73907 (7)	0.6562 (3)	0.4342 (3)	0.0162 (3)	
H3O	0.7155 (13)	0.558 (5)	0.494 (5)	0.023 (6)*	
H4O	0.7272 (17)	0.641 (6)	0.322 (7)	0.041 (9)*	
O7	0.80241 (7)	1.0903 (2)	0.4929 (2)	0.0140 (3)	
H5O	0.7656 (14)	1.115 (5)	0.421 (5)	0.024 (7)*	
H6O	0.8277 (13)	1.189 (5)	0.471 (6)	0.023 (7)*	
C1	0.91739 (8)	0.3990 (3)	0.2095 (3)	0.0128 (4)	
C2	0.92180 (9)	0.5614 (3)	0.0138 (3)	0.0177 (4)	
H2A	0.9595	0.6678	0.0394	0.021*	
H2B	0.9326	0.4735	−0.1198	0.021*	
C3	0.85780 (9)	0.6960 (3)	−0.0275 (3)	0.0124 (3)	

### Atomic displacement parameters (Å^2^)

**Table:**

	*U*^11^	*U*^22^	*U*^33^	*U*^12^	*U*^13^	*U*^23^
Mg1	0.0125 (3)	0.0134 (3)	0.0053 (3)	0.0000 (2)	0.0001 (2)	0.0004 (2)
O1	0.0191 (6)	0.0153 (6)	0.0097 (7)	0.0016 (5)	0.0040 (5)	0.0016 (5)
O2	0.0192 (6)	0.0199 (7)	0.0182 (8)	0.0055 (5)	0.0062 (6)	0.0022 (6)
O3	0.0225 (7)	0.0229 (8)	0.0053 (6)	0.0001 (5)	−0.0014 (5)	0.0012 (6)
O4	0.0175 (7)	0.0187 (7)	0.0072 (7)	0.0033 (5)	0.0002 (5)	0.0009 (6)
O5	0.0163 (6)	0.0232 (7)	0.0245 (8)	−0.0034 (6)	−0.0070 (6)	0.0103 (7)
O6	0.0176 (6)	0.0225 (7)	0.0085 (7)	−0.0062 (5)	−0.0014 (5)	0.0018 (6)
O7	0.0143 (6)	0.0146 (6)	0.0131 (7)	−0.0006 (5)	0.0012 (5)	0.0008 (5)
C1	0.0122 (7)	0.0158 (9)	0.0104 (8)	−0.0011 (6)	−0.0004 (6)	0.0011 (7)
C2	0.0169 (8)	0.0253 (10)	0.0110 (9)	0.0053 (7)	0.0046 (7)	0.0061 (8)
C3	0.0144 (8)	0.0164 (8)	0.0065 (8)	−0.0014 (6)	0.0018 (7)	0.0030 (7)

### Geometric parameters (Å, º)

**Table:**

Mg1—O3^i^	2.0323 (18)	O5—H1O	0.91 (3)
Mg1—O5	2.0377 (15)	O5—H2O	0.85 (4)
Mg1—O4	2.0700 (16)	O6—H3O	0.83 (3)
Mg1—O6	2.0706 (15)	O6—H4O	0.73 (4)
Mg1—O1	2.0725 (15)	O7—H5O	0.86 (3)
Mg1—O7	2.1273 (14)	O7—H6O	0.78 (3)
O1—C1	1.270 (2)	C1—C2	1.535 (3)
O2—C1	1.244 (2)	C2—C3	1.519 (2)
O3—C3	1.261 (3)	C2—H2A	0.9900
O3—Mg1^ii^	2.0323 (18)	C2—H2B	0.9900
O4—C3	1.259 (3)		
			
O3^i^—Mg1—O5	94.40 (7)	H1O—O5—H2O	117 (3)
O3^i^—Mg1—O4	171.80 (6)	Mg1—O6—H3O	134.5 (19)
O5—Mg1—O4	93.70 (7)	Mg1—O6—H4O	115 (3)
O3^i^—Mg1—O6	86.35 (6)	H3O—O6—H4O	98 (3)
O5—Mg1—O6	172.19 (7)	Mg1—O7—H5O	114 (2)
O4—Mg1—O6	85.46 (6)	Mg1—O7—H6O	117.8 (19)
O3^i^—Mg1—O1	96.53 (6)	H5O—O7—H6O	109 (3)
O5—Mg1—O1	92.20 (6)	O2—C1—O1	123.80 (17)
O4—Mg1—O1	84.41 (6)	O2—C1—C2	116.45 (17)
O6—Mg1—O1	95.45 (6)	O1—C1—C2	119.75 (15)
O3^i^—Mg1—O7	94.16 (7)	C3—C2—C1	114.27 (14)
O5—Mg1—O7	85.33 (7)	C3—C2—H2A	108.7
O4—Mg1—O7	85.25 (7)	C1—C2—H2A	108.7
O6—Mg1—O7	86.86 (6)	C3—C2—H2B	108.7
O1—Mg1—O7	169.19 (7)	C1—C2—H2B	108.7
C1—O1—Mg1	128.87 (12)	H2A—C2—H2B	107.6
C3—O3—Mg1^ii^	145.80 (12)	O4—C3—O3	122.26 (16)
C3—O4—Mg1	128.60 (13)	O4—C3—C2	118.72 (17)
Mg1—O5—H1O	129.3 (19)	O3—C3—C2	119.02 (17)
Mg1—O5—H2O	112.6 (19)		
			
Mg1—O1—C1—O2	160.10 (14)	Mg1—O4—C3—C2	−30.2 (2)
Mg1—O1—C1—C2	−19.6 (2)	Mg1^ii^—O3—C3—O4	115.7 (2)
O2—C1—C2—C3	150.96 (18)	Mg1^ii^—O3—C3—C2	−65.3 (3)
O1—C1—C2—C3	−29.3 (3)	C1—C2—C3—O4	55.5 (2)
Mg1—O4—C3—O3	148.73 (14)	C1—C2—C3—O3	−123.53 (19)

Symmetry codes: (i) *x*, *y*, *z*+1; (ii) *x*, *y*, *z*−1.

### Hydrogen-bond geometry (Å, º)

**Table:**

*D*—H···*A*	*D*—H	H···*A*	*D*···*A*	*D*—H···*A*
O5—H1*O*···O2^iii^	0.91 (3)	1.80 (3)	2.701 (2)	170 (3)
O5—H2*O*···O2^iv^	0.85 (4)	1.85 (4)	2.678 (2)	165 (3)
O6—H3*O*···O4^v^	0.83 (3)	1.93 (3)	2.759 (2)	175 (3)
O6—H4*O*···O7^vi^	0.73 (4)	2.11 (4)	2.838 (2)	177 (3)
O7—H5*O*···O3^vii^	0.86 (3)	2.11 (3)	2.963 (2)	172 (3)
O7—H6*O*···O1^iv^	0.78 (3)	1.93 (3)	2.703 (2)	169 (3)

Symmetry codes: (iii) −*x*+2, −*y*+1, *z*+1/2; (iv) *x*, *y*+1, *z*; (v) −*x*+3/2, *y*−1/2, *z*+1/2; (vi) −*x*+3/2, *y*−1/2, *z*−1/2; (vii) −*x*+3/2, *y*+1/2, *z*+1/2.
